# The clinical efficacy of percutaneous endoscopic lumbar discectomy combined with platelet-rich plasma injection for lumbar disc herniation: a systematic review and meta-analysis

**DOI:** 10.3389/fsurg.2025.1601772

**Published:** 2025-05-27

**Authors:** Hua Song, Ying Zhang

**Affiliations:** ^1^ Department of Orthopaedics, Tengzhou Central People’s Hospital, Tengzhou, Shandong, China; ^2^ Department of Orthopaedics, People’s Liberation Army Joint Logistic Support Force 920th Hospital, Kunming, Yunnan, China

**Keywords:** platelet-rich plasma, percutaneous endoscopic lumbar discectomy, lumbar disc herniation, clinical efficacy, pain measurement, meta-analysis

## Abstract

**Objective:**

Although percutaneous endoscopic lumbar discectomy (PELD) has shown favorable outcomes in the treatment of LDH patients, the issue of recurrence caused by potential disc degeneration remains unresolved. Regenerative therapy with platelet-rich plasma (PRP) injection offers the potential to reduce recurrence rates and improve clinical outcomes. This systematic review and meta-analysis evaluated the clinical efficacy of combining PELD with PRP injection as a novel therapeutic approach for LDH.

**Methods:**

A comprehensive literature search was conducted in the PubMed, Embase, Web of Science, and Cochrane databases, with the search period ending on October 30, 2024. Data were extracted and analyzed to evaluate recurrence rates, pain relief, functional outcomes, and intervertebral disc health status.

**Results:**

A total of 4 eligible studies were identified in this research, comprising 421 patients, of whom 212 received the combined treatment of PRP and PELD, while 209 underwent PELD alone. The results demonstrated that the combined PELD and PRP therapy significantly reduced recurrence rates (OR: 0.21, 95% CI: 0.07 to 0.64, *p* = 0.006) and improved VAS pain scores for both back and leg pain at specific follow-up time. Additionally, intervertebral disc height at the final follow-up was significantly greater in the combined PELD and PRP group (MD: 0.88, 95% CI: 0.57 to 1.20, *p* < 0.00001), indicating the potential of the combined therapy to restore degenerative discs.

**Conclusions:**

The study indicates that PELD combined with PRP therapy provides better clinical outcomes compared to PELD alone, particularly in reducing recurrence rates, alleviating pain, and improving functional recovery. However, future studies with larger sample sizes and extended follow-up durations are warranted to validate the long-term efficacy and safety of this innovative therapeutic approach.

**Systematic Review Registration:**

https://www.crd.york.ac.uk/PROSPERO/view/CRD42024621150, PROSPERO CRD42024621150.

## Introduction

Lumbar disc herniation (LDH) is a relatively common spinal disorder and has become a leading cause of lower back and leg pain (1). With the trend of younger onset of LDH, its social and clinical impact has drawn widespread attention (2). For patients with mild symptoms, conservative treatment can lead to recovery within 1–3 months (3). However, recurrent and severe LDH eventually requires surgical intervention to resolve persistent symptoms and prevent recurrence (4). Traditional open surgery achieves favorable clinical outcomes by completely removing the calcified intervertebral disc. However, laminectomy and extensive dissection of the paraspinal muscles can result in significant tissue damage and complications (5, 6).

Advances in minimally invasive spinal surgery have popularized percutaneous endoscopic lumbar discectomy (PELD). Compared to traditional open surgery, PELD offers shorter recovery times and better preservation of spinal anatomy, largely maintaining lumbar spinal stability (7, 8). Despite its advantages, PELD has been associated with intraoperative nerve-related complications, primarily including postoperative numbness and pain (9). On the other hand, PELD focuses mainly on neural decompression, leaving underlying degenerative changes within the disc unaddressed, which may limit long-term outcomes and predispose patients to recurrence. To reduce recurrence rates and improve clinical efficacy, a growing number of researchers are focusing on regenerative treatments for intervertebral discs (10, 11).

The integration of regenerative therapies has opened new avenues for the treatment of degenerative spinal diseases (12). For instance, platelet-rich plasma (PRP), with its high concentration of growth factors that stimulate tissue repair, regulate inflammation, and support disc remodeling (13, 14), has emerged as a promising adjuvant therapy. Mechanistically, PRP can drive tissue repair and regeneration by regulating the release of growth factors and binding to extracellular receptors on target cells, thereby promoting cell differentiation, proliferation, and migration (15). The healing potential of PRP has been demonstrated across various medical fields, and its application in spinal diseases, particularly as a combined therapy with surgical techniques such as PELD, is gaining increasing attention from clinicians (16). By potentially promoting annular repair and reducing inflammation, PRP combined with minimally invasive discectomy holds promise for improving the long-term clinical outcomes of LDH and reducing recurrence rates. However, current evidence evaluating the significant advantages of the combined use of PELD and PRP remains limited and fragmented, with existing studies often constrained by small sample sizes or methodological inconsistencies. Furthermore, there is a lack of evidence assessing the short-, medium-, and long-term clinical efficacy of the combined PELD and PRP therapy.

This systematic review and meta-analysis aims to systematically assess whether PELD combined with PRP injection improves clinical outcomes compared to PELD alone in patients with LDH, with specific focus on pain relief, functional recovery, recurrence rates, and intervertebral disc health.

## Materials and methods

### Search strategy

We performed comprehensive literature searches in PubMed, Embase, Web of Science, and Cochrane databases, covering publications from their inception to 30 October 2024. The following keyword combinations were utilized: (“percutaneous endoscopic lumbar discectomy” OR “PELD” OR “endoscopic discectomy” OR “platelet-rich plasma” OR “PRP” OR “platelet concentrates”) AND (“lumbar disc herniation” OR “LDH” OR “herniated lumbar disc” OR “lumbar intervertebral disc herniation”). Boolean operators (including OR, AND, NOT) were used to ensure both comprehensiveness and precision in the search terms. The complete search strategy for PubMed is provided in Supplementary Table S1. Two authors independently screened the retrieved literature and evaluated it against the inclusion criteria based on titles and/or abstracts. Discrepancies were resolved through discussion with a third senior author. Full-text articles meeting the inclusion criteria were comprehensively reviewed, and their references were manually examined to ensure the inclusion of all relevant studies. This meta-analysis was conducted in accordance with the Preferred Reporting Items for Systematic Reviews and Meta-Analyses (PRISMA) guidelines (17, 18). The study was registered with the International Prospective Register of Systematic Reviews (PROSPERO) under the ID CRD42024621150 prior to initiating the database search and study selection process.

### Inclusion and exclusion criteria

Inclusion criteria:

1) The study subjects were adult patients diagnosed with lumbar disc herniation (LDH) who met the diagnostic criteria based on clinical symptoms and imaging examinations (e.g., MRI or CT);
2) The experimental group received PELD combined with PRP injection therapy;
3) Included clinical evidence consisted of randomized controlled trials (RCTs), prospective cohort studies, retrospective cohort studies, and case-control studies;
4) Only English-language literature was included;
5) The studies were required to report at least one primary clinical outcome measure, such as recurrence rate, pain in the back and legs, the Macnab criteria, Pfirrmann grade, pain relief (e.g., VAS score), or functional improvement (e.g., ODI score).

Exclusion criteria:

1) Reviews, letters, abstracts, commentaries, case reports, and studies that are not case-control studies;
2) Preclinical studies based on cell models, animal models, or cadaveric research were excluded;
3) Studies with fewer than 10 cases in either the experimental group or the control group;
4) Literature with significant confounding factors (e.g., patients with major comorbidities that affect the evaluation of treatment outcomes) or flaws in study design.

### Study selection and data extraction

Two independent authors (Hua Song and Ying Zhang) screened titles and abstracts. The extracted information primarily included the first author, year of publication, study design, age, gender, sample size, follow-up duration, PRP injection volume, pain and functional scores at different follow-up time points, and primary outcomes (number of recurrence cases and complications). The summarized data were reviewed and verified by a third author to ensure accuracy.

### Quality assessment

This study included one RCT and three retrospective cohort studies, with the quality of the RCT assessed using The Cochrane Collaboration's tool (19) and the retrospective cohort studies evaluated using the Newcastle-Ottawa Scale (NOS). Specifically, The Cochrane Collaboration's tool evaluates seven aspects: random sequence generation, allocation blinding, blinding of participants, blinding of outcome measures, incomplete outcome data, selective reporting, and other biases. The judgment of risk of bias is expressed using three categories: “low risk”, “high risk”, and “unclear risk”. A NOS score of ≥7 was considered low risk of bias, a score of 4–6 indicated moderate risk of bias, and a score <4 was classified as high risk of bias. Two independent and experienced authors rated the studies, and final scores were determined through discussion with a senior third author.

### Outcomes of interest

#### Primary outcomes

1) Recurrence rate at the follow-up endpoint;
2) VAS scores for back and leg pain, and ODI score (3 days, 3 months, 6 months and 12 months);

#### Secondary outcomes

1) The Macnab criteria (Excellent, Fair, Good) and Pfirrmann grade (Ⅱ, Ⅲ, Ⅳ);
2) The Japanese Orthopaedic Association (JOA) score;
3) Final follow-up intervertebral disc height.

### Statistical analysis

This study used the *I*^2^ statistic and *χ*^2^ test to evaluate heterogeneity. According to the Cochrane Handbook, heterogeneity was classified as follows: 0%−40% as low heterogeneity, 30%−60% as moderate heterogeneity, 50%−90% as substantial heterogeneity, and 75%−100% as considerable heterogeneity. When *I*^2^ ≤ 50% and *p* > 0.10, the combined data were considered to have no significant heterogeneity, and a fixed-effect model was applied. Otherwise, a random-effects model was used for pooled effect analysis (20). Differences in dichotomous variables, such as recurrence rate and the Macnab criteria, were analyzed by calculating the odds ratio (OR), while continuous variables were evaluated by calculating the mean difference (MD), both with 95% confidence intervals (CI) (21). Subgroup analyses were conducted based on the time points for each outcome measure. We planned to assess publication bias using funnel plots and Egger's test if more than 10 studies were included for any outcome. Sensitivity analyses were planned to assess the impact of excluding studies with high risk of bias. All data analyses were performed using RevMan version 5.4 software (The Cochrane Collaboration, Copenhagen, Denmark). A *p*-value < 0.05 was considered statistically significant.

## Results

### Literature search

**Figure 1:**
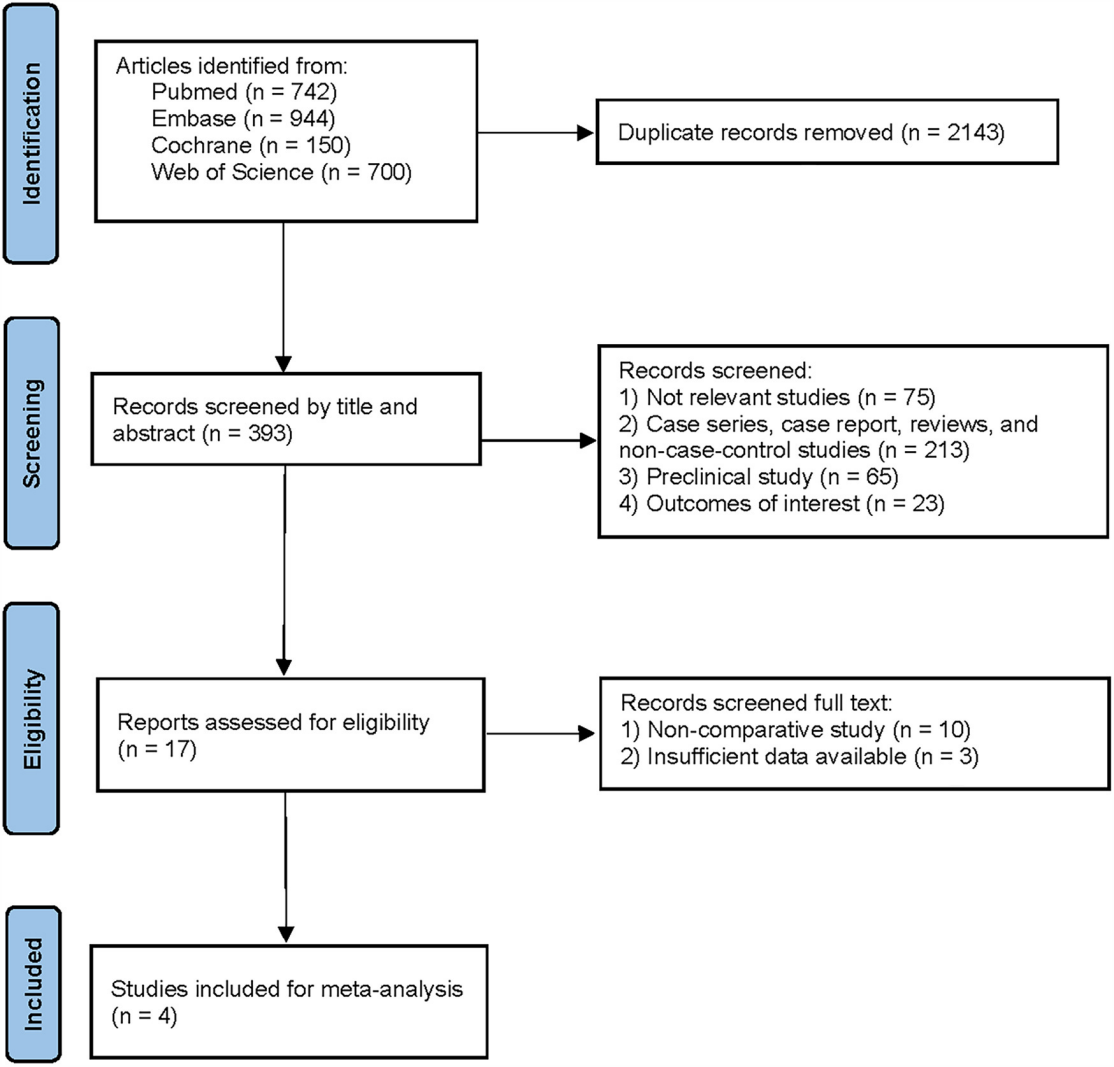
Flowchart of the study based on the PRISMA guidelines.

A comprehensive search of four major electronic databases (PubMed, Embase, Web of Science, and Cochrane databases) initially identified 2,536 articles. After independent screening by one author, 2,143 duplicate articles were manually removed. Subsequently, the titles and abstracts of the remaining articles were reviewed, resulting in the exclusion of an additional 376 articles. Finally, 17 articles were subjected to full-text review, and 4 articles meeting the eligibility criteria were selected for further data analysis. The PRISMA flow diagram for this study is presented in Figure 1.

### Study characteristics

Table 1 provides detailed information on the study characteristics of the included articles. In terms of study design, all studies were retrospective cohort studies (22–24) except for one (25), which was an RCT. Overall, the studies were published between 2022 and 2024, with patient data originating exclusively from China. A total of 421 patients were included in the data analysis, of which 212 received PELD combined with PRP treatment, while the remaining 209 underwent PELD treatment alone. Specifically, the patients' age range was 31.7–58.35 years, BMI ranged from 18.2 to 28.27, and disease duration ranged from 1.19 to 31.94 months. Surgical levels were concentrated in L3/4, L4/5, and L5/S1. For PRP, the total blood volume used ranged from 18 to 50 ml, with an injection volume of 4–5 ml. Regarding outcome measures, three of the studies reported complications. Two studies comprehensively reported VAS scores for back and leg pain at four follow-up time points, all four studies reported ODI and VAS scores for low back pain at three months, three studies reported the Macnab criteria, and three studies reported the Pfirrmann grade.

**Table 1:** Characteristics of articles included in the meta-analysis.

Author	Year	Country	Study design	Age (treatment vs. control, Years ± SD, range)	Number of female/male (treatment vs. control)	BMI (kg/m2) (treatment vs. control)	Duration of disease (treatment vs. control, month ± SD, range)	Affected levels (treatment vs. control)			Platelet (×10^9^/L) (treatment vs. control)	PRP preparation method	Volume of whole blood used	Injectate volume
								L3/4	L4/5	L5/S1				
Li et al. (22)	2024	China	Retrospective cohort	43.61 ± 11.72 vs. 44.25 ± 11.56	36/39 vs. 34/46	24.22 ± 2.98 vs. 24.41 ± 3.09	21.87 ± 7.86 vs. 23.54 ± 8.40	10 vs. 12	36 vs. 40	29 vs. 28	211.25 ± 28.84 vs. 214.30 ± 30.29	Sterile WEGO PRP kit	36 ml	4.0 ml
Zhang et al. (23)	2023	China	Retrospective cohort	41.5 ± 9.8 vs. 45.8 ± 11.0	22/28 vs. 22/26	21.3 ± 3.1 vs. 23.4 ± 2.9	12.1 ± 10.1 vs. 15.3 ± 8.5	9 vs. 8	18 vs. 16	23 vs. 24	212.9 ± 48.1 vs. 228.3 ± 52.3	PRP preparation kit	30 ml	3.5–4.0 ml
Jiang et al. (24)	2022	China	Prospective cohort	48.1 ± 10.25 vs. 45.9 ± 9,83	24/33 vs. 19/32	No description	No description	6 vs. 7	33 vs. 30	12 vs. 20	217.0 ± 52.1 vs. 235.9 ± 65.9	Sterile WEGO PRP kit	50 ml	4.0 ml
Qi et al. (25)	2024	China	Randomized controlled trial (RCT)	44.20 ± 7.32 vs. 43.30 ± 6,26	14/16 vs. 12/18	25.89 ± 1.64 vs. 26.54 ± 1.73	5.98 ± 4.46 vs. 6.40 ± 5.21	5 vs. 4	14 vs. 13	11 vs. 13	No description	Harvest centrifuge	18 ml	3.0 ml

### Quality assessment

Two independent authors meticulously rated the four included studies, with any discrepancies resolved through discussion with a third researcher. The RCT explicitly reported using a random number table for randomization. Apart from unclear risk for allocation concealment and blinding of outcome assessment, all other domains were rated as low risk of bias (Supplementary Figure S1). Two cohort studies received an NOS score of 9, and one study received a score of 8. All studies were evaluated as having a low risk of bias (Supplementary Table S2).

## Clinical efficacy evaluation

### Recurrence rate

**Figure 2:**
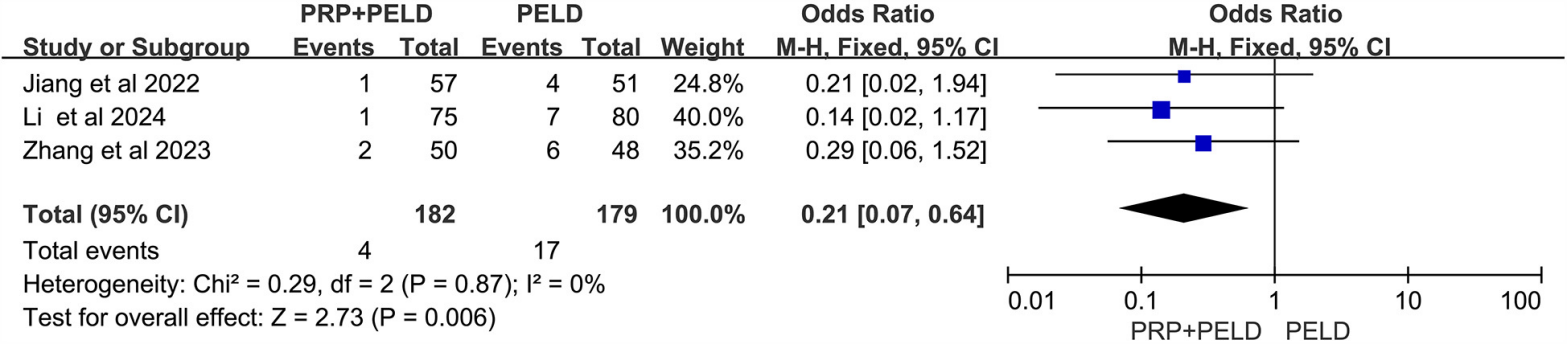
Meta-analysis results of the overall recurrence rate.

To investigate whether PELD combined with PRP treatment offers a significant advantage over traditional PELD surgery in reducing the overall recurrence rate in patients with lumbar disc herniation, data on recurrence cases from three relevant studies were collected. The heterogeneity test results (*I*^2^ < 50%, *p* = 0.87) indicated no significant heterogeneity among the studies. The pooled data from these three studies demonstrated that the recurrence rate was significantly lower in the PRP + PELD group compared to the PELD-only group (OR: 0.21, 95% CI: 0.07 to 0.64, *p* = 0.006) (Figure 2).

### VAS scores

**Figure 3:**
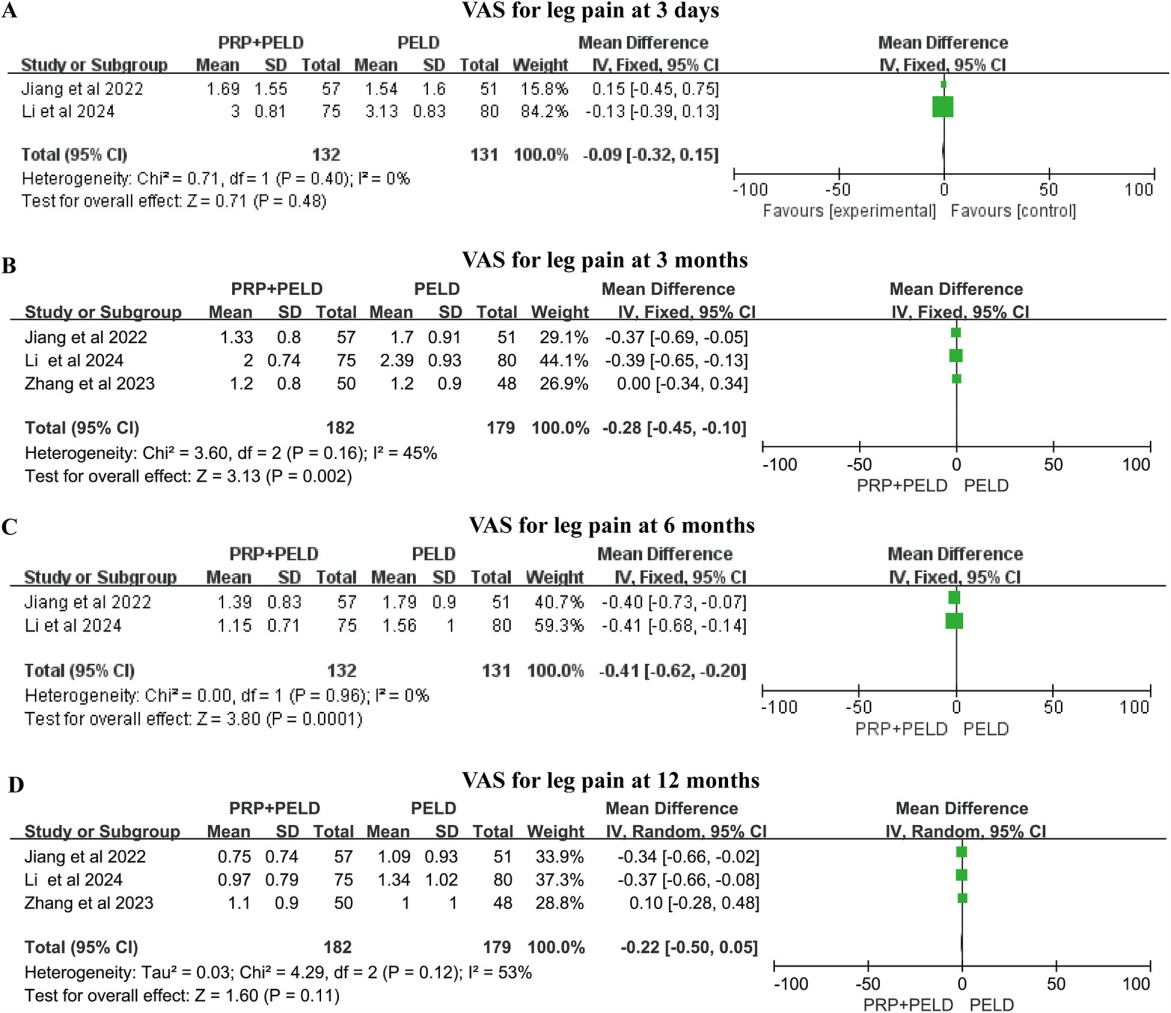
Meta-analysis results of VAS scores for leg pain at different follow-up time. **(A)** VAS scores for leg pain at 3 days; **(B)** VAS scores for leg pain at 3 months; **(C)** VAS scores for leg pain at 6 months; **(D)** VAS scores for leg pain at 12 months.

The VAS score for low back pain is commonly used in clinical practice to assess the rehabilitation status of patients after lumbar surgery. All four included studies utilized this measure to evaluate pain outcomes for back and leg pain at different follow-up time points (3 days, 3 months, 6 months, and 12 months) (Figures 3A–D). For the assessment of leg pain, the pooled results demonstrated that the PRP + PELD group was associated with significantly lower pain levels at 3 months (short-term period) (MD: −0.28, 95% CI: −0.45 to −0.10, *p* = 0.002) and 6 months (mid-term period) (MD: −0.41, 95% CI: −0.62 to −0.20, *p* = 0.0001). However, pooled analyses for 3 days (shorter-term period) and 12 months (long-term period) showed no significant differences in leg pain scores between the groups.

**Figure 4:**
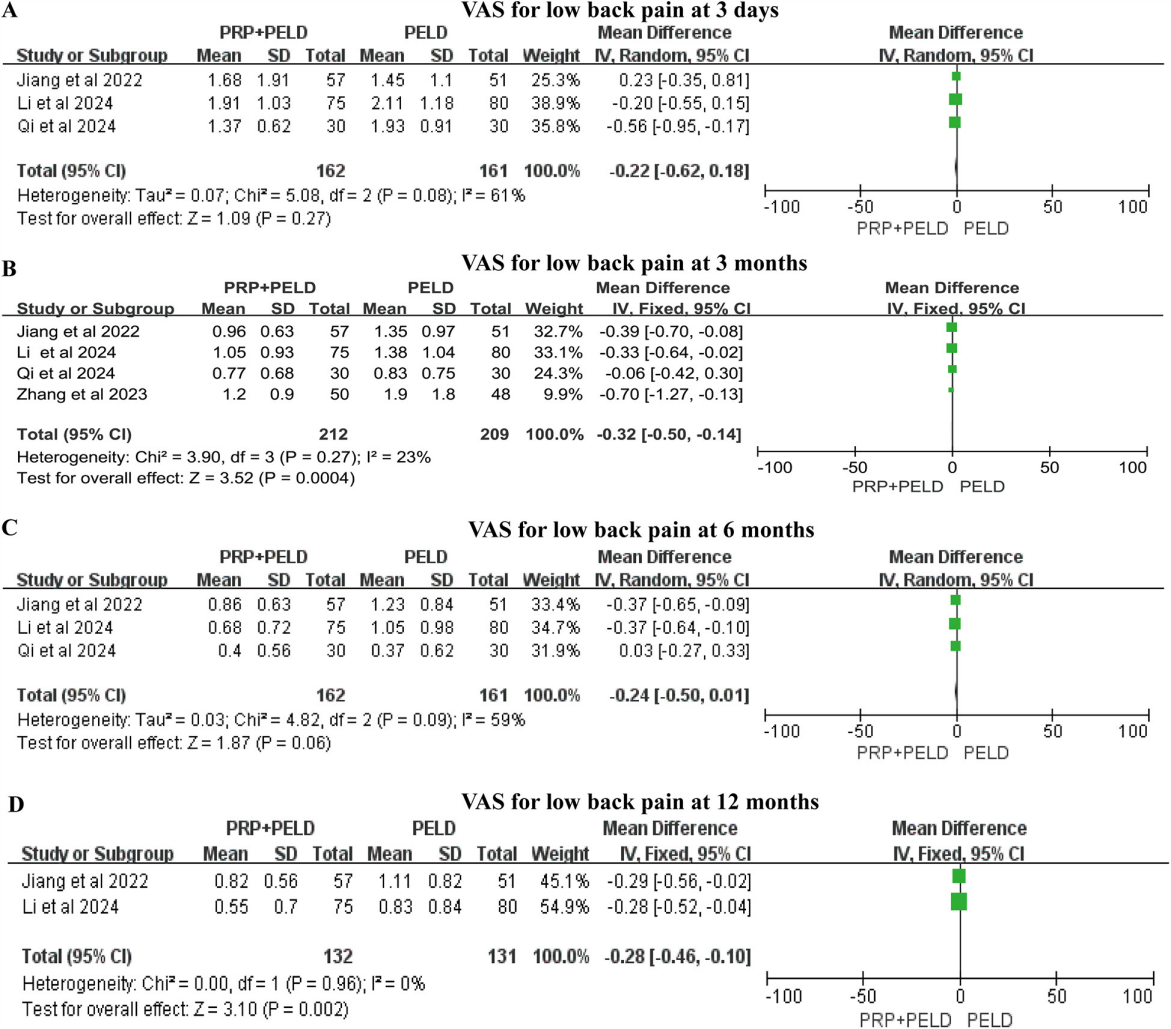
Meta-analysis results of VAS scores for low back pain at different follow-up time. **(A)** VAS scores for low back pain at 3 days; **(B)** VAS scores for low back pain at 3 months; **(C)** VAS scores for low back pain at 6 months; **(D)** VAS scores for low back pain at 12 months.

For low back pain, the pooled results indicated that the PRP + PELD group was associated with significantly lower pain levels at the short-term period (3 months) (MD: −0.32, 95% CI: −0.50 to −0.14, *p* = 0.0004) and longer-term period (12 months) (MD: −0.28, 95% CI: −0.46 to −0.10, *p* = 0.002) follow-up time points. However, no significant differences in the VAS score for low back pain were observed between the two groups at shorter-term (3 days) and mid-term (6 months) evaluations (Figures 4A–D). Given the variations in PRP preparation and intervention across studies, a subgroup analysis of the 3-month VAS score for low back pain was conducted based on preparation method, total blood volume, and injection volume. The results indicated that, compared with the PELD-only group, the PRP preparation kits subgroup, total blood volume >30 ml subgroup, and an injection volume of 4.0 ml subgroup may exert a beneficial effect in reducing VAS scores for low back pain. However, due to the relatively small sample sizes within these subgroups, these findings should be interpreted with caution (Supplementary Figure S2).

### ODI score

**Figure 5:**
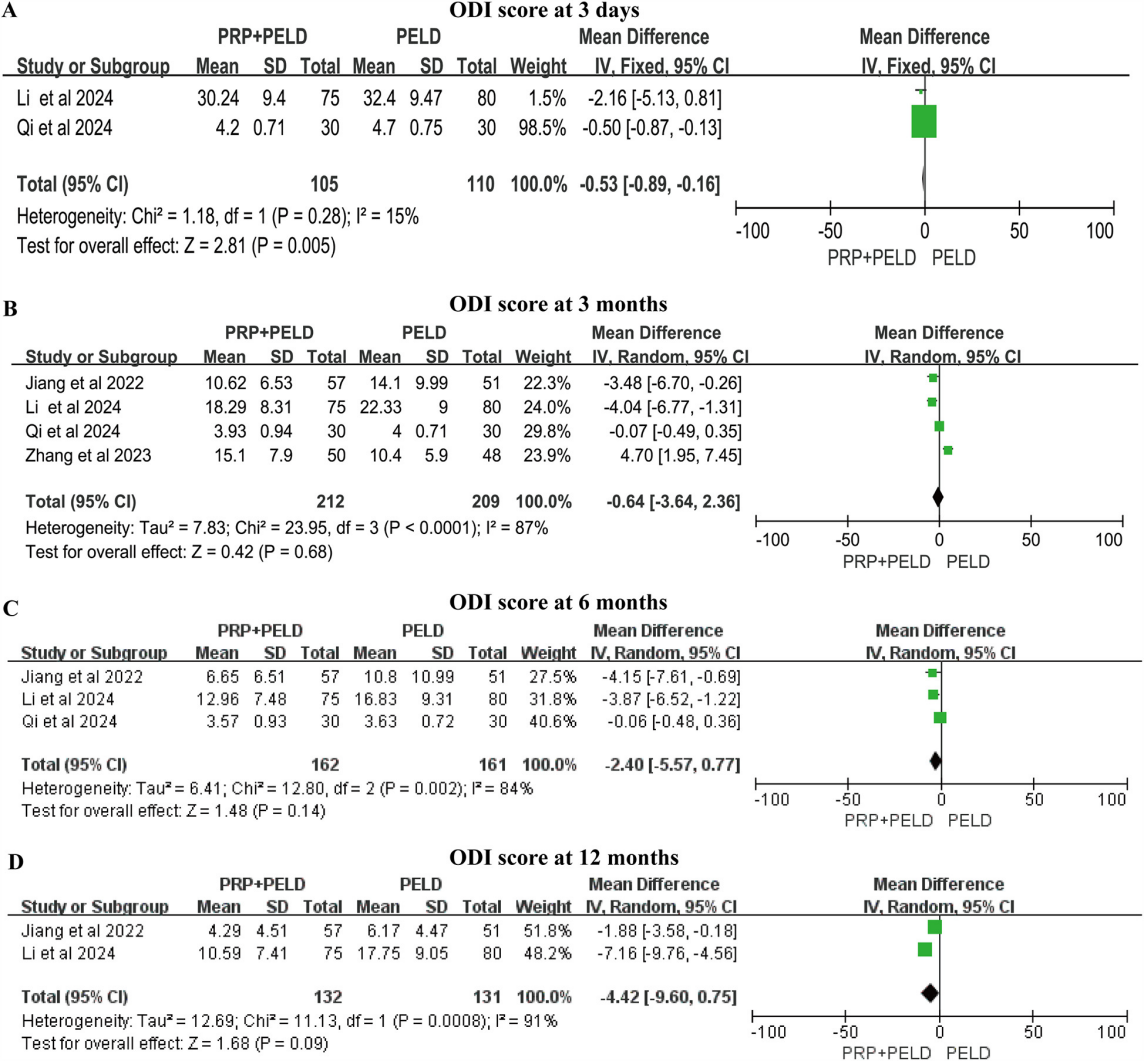
Meta-analysis results of ODI scores at different follow-up time. **(A)** ODI score at 3 days; **(B)** ODI score at 3 months; **(C)** ODI score at 6 months; **(D)** ODI score at 12 months.

Although the follow-up time points varied, all four studies reported relevant data on the ODI score. In the shorter-term period (3 days), the pooled results indicated that the PRP + PELD group was associated with a significantly lower functional disability index (MD: −0.53, 95% CI: −0.89 to −0.16, *p* = 0.005). However, at 3 months, 6 months, and 12 months, no significant differences in ODI scores were observed between the two groups (Figures 5A–D). The subgroup analysis suggested that the total blood volume >30 ml subgroup and the 4.0 ml injection volume subgroup may have a positive effect on improving the 3-month ODI score. However, due to limitations in the number of studies and sample sizes, these findings should be interpreted with caution (Supplementary Figure S3).

### The Macnab criteria

**Figure 6:**
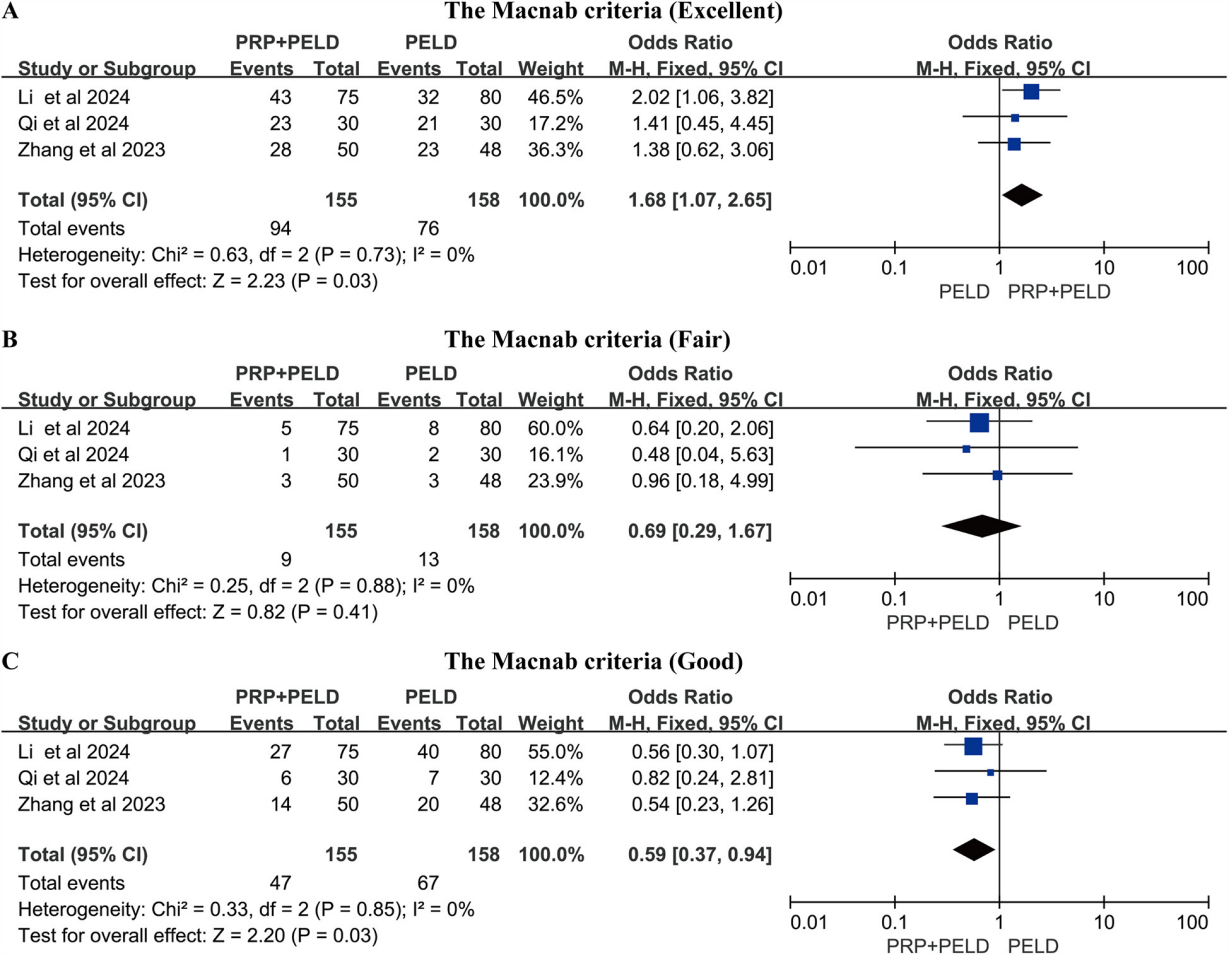
Meta-analysis results based on the Macnab criteria. **(A)** Macnab criteria (Excellent); **(B)** Macnab criteria (Fair); **(C)** Macnab criteria (Good).

A total of three studies reported the Macnab criteria for postoperative patients, and we conducted pooled analyses of the data classified as “excellent”, “good” and “fair”. The results of the pooled analysis showed that the PRP + PELD group had a significantly higher rate of “excellent” outcomes (MD: 1.68, 95% CI: 1.07 to 2.65, *p* = 0.03), while the PELD group had a higher rate of “good” outcomes (MD: 0.59, 95% CI: 0.37 to 0.94, *p* = 0.03) (Figures 6A–C).

### The pfirrmann grade

**Figure 7:**
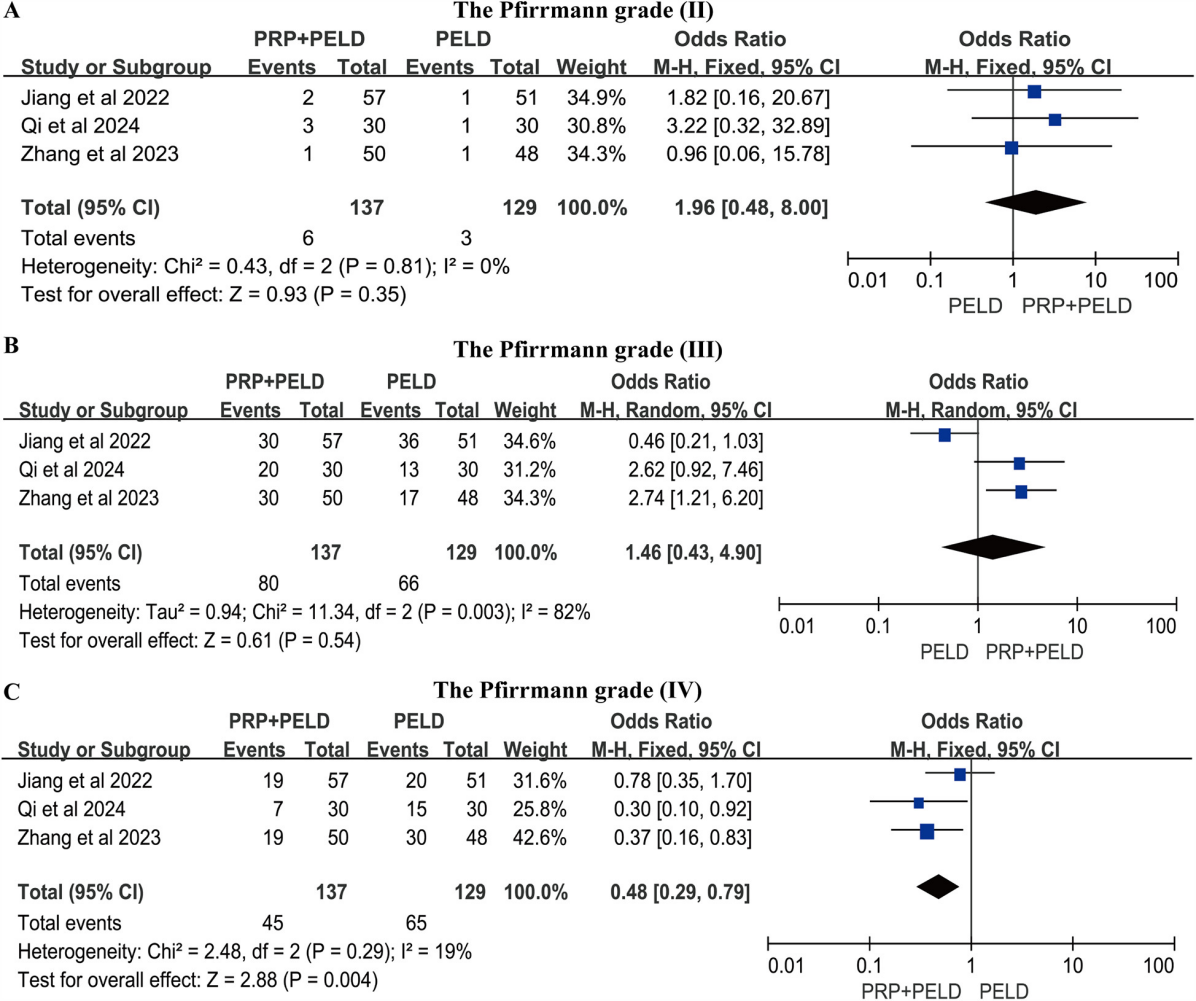
Meta-analysis results of postoperative pfirrmann grade. **(A)** The Pfirrmann grade (Ⅱ); **(B)** The Pfirrmann grade (Ⅲ); **(C)** The Pfirrmann grade (Ⅳ).

Three studies reported the Pfirrmann grade outcomes for a total of 266 postoperative patients. The pooled analysis results showed that the PRP + PELD group had significantly fewer patients with Pfirrmann grade IV degeneration postoperatively (OR: 0.48, 95% CI: 0.29 to 0.79, *p* = 0.004) (Figures 7A–C), suggesting a potential improvement in disc degeneration.

### JOA score and final follow-up intervertebral disc height

**Figure 8:**
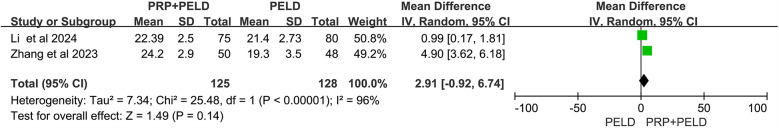
Meta-analysis results of JOA score.

**Figure 9:**
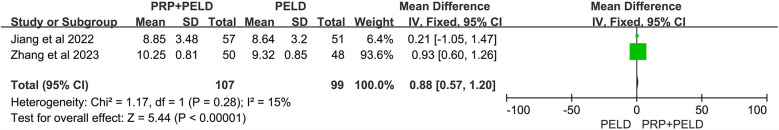
Meta-analysis results of intervertebral disc height at final follow-up.

In the evaluation of the JOR score, no significant differences were observed between the two groups (Figure 8). Interestingly, in the analysis of disc height at the final follow-up, the pooled results showed that the PRP + PELD group was associated with significantly greater disc height (MD: 0.88, 95% CI: 0.57 to 1.20, *p* < 0.00001) (Figure 9).

## Discussion

To the best of our knowledge, this meta-analysis is the first comprehensive evaluation of the clinical efficacy of PELD combined with intradiscal PRP injection for the treatment of LDH, analyzing its effects in terms of pain relief, functional recovery, and recurrence outcomes. The study indicated that PELD combined with PRP therapy provided better clinical outcomes compared to PELD alone. PRP has been shown in both *in vitro* and *in vivo* experiments to promote intervertebral disc cell regeneration, support neural function recovery, and downregulate the expression of inflammatory factors (26, 27). Intradiscal PRP injection holds promise in synergizing with surgery to alleviate symptoms while reducing the risk of recurrence after LDH surgery.

The main findings of this meta-analysis indicate that the recurrence rate during follow-up in LDH patients treated with PELD combined with PRP injection was significantly lower than in those treated with PELD alone, highlighting the effectiveness of incorporating PRP to improve clinical outcomes in LDH patients. In an earlier preliminary clinical trial, Akeda et al. (28) reported that intradiscal PRP injection for LDH patients was both safe and feasible. In a subsequent long-term follow-up study lasting up to 5.9 years, the same team demonstrated that 91% of patients experienced significant improvements in VAS and disability scores following intradiscal PRP injection (29). Furthermore, numerous researchers have confirmed the clinical efficacy of PRP in alleviating discogenic low back pain. Another long-term follow-up study, spanning 5–9 years, showed that PRP injection significantly improved pain and functional outcomes in patients with low back pain (30). A recent single-arm meta-analysis demonstrated the beneficial effects of intradiscal PRP injection on pain relief outcomes, with evaluation metrics including VAS and the Short Form Health Survey (SF-36) (31). In our pooled analysis, LDH patients treated with the combined PELD and PRP therapy exhibited significant pain improvement at both 3 months and 12 months, demonstrating the short- and long-term effectiveness of PRP in relieving pain. However, for VAS scores at 3 days and 6 months, only two studies were available, limiting the interpretability of these results.

In addition to alleviating pain, PRP has been shown to improve functional impairment in LDH patients. Centeno et al. (32) investigated the clinical efficacy of transforaminal epidural PRP injections in patients with lumbar radicular pain and found significant improvements in pain and functional impairment compared to baseline after a two-year follow-up. Similarly, Le et al. (33) reported a significant reduction in VAS and ODI scores in LDH patients following transforaminal PRP injections, with no adverse events observed. Jain et al. (34) found a positive correlation between platelet concentration in PRP and ODI scores in low back pain patients through a prospective clinical trial. Another study compared the efficacy of steroid injections and PRP injections for lumbar radicular pain, revealing similar clinical outcomes (in terms of pain and functional assessments), suggesting that PRP could serve as an alternative to steroids (35). In our study, pooled analysis demonstrated that the combined therapy of PELD and PRP injections significantly reduced disability indices, and the Macnab criteria showed a higher rate of excellent outcomes, indicating that the addition of PRP plays a positive role in improving functional outcomes in LDH patients.

Furthermore, intervertebral disc degeneration in LDH patients is accompanied by upregulation of pro-inflammatory factors and loss of extracellular matrix (36). Previous animal and *in vitro* cell experiments have validated the effects and potential mechanisms of PRP on disc degeneration. Gullung et al. (37) demonstrated that PRP administration exerted protective effects on damaged L4-L5 discs in rats, emphasizing the critical role of PRP in early intervertebral disc degeneration. In an *in vitro* organ culture system of degenerative discs, PRP was shown to promote nucleus pulposus regeneration and participate in cartilage formation, with a significant increase in the disc height index (38). Changes in disc height are an important indicator of the therapeutic efficacy of PRP injections for disc degeneration. In a rabbit model of disc degeneration, Obata et al. (39) observed that PRP facilitated the restoration of disc height, accompanied by an increase in the number of chondrocytes. Furthermore, a meta-analysis of animal studies revealed that PRP treatment significantly restored disc height and reduced histological degeneration grades (40). Consistent with these findings, our meta-analysis also showed a significant increase in disc height at the final follow-up in the PELD combined with PRP injection group, demonstrating its potential to restore degenerative discs.

The results of this meta-analysis highlighted the clinical efficacy of combining PELD with PRP injection in the treatment of patients with LDH, providing valuable evidence to support future clinical trials. Moreover, this innovative combined therapy may also influence clinical decision-making by encouraging clinicians to reconsider sole surgical interventions in favor of more integrated treatment approaches. However, this meta-analysis has several limitations. First, despite a comprehensive search and screening of four major databases, only four studies met the inclusion criteria for this emerging combination therapy, which may affect the robustness of the analysis results. Second, the follow-up time points in the included studies were not consistent, resulting in only two studies providing data for some specific time points in the pooled analysis. Third, the overall quality of evidence from all included studies was low. Future research should focus on large-sample, multicenter, and prospective RCTs to enhance the strength and reliability of the conclusions. Moreover, although the injection method of PRP was generally consistent, there were varying degrees of differences in PRP preparation (including preparation methods and blood volume) and dosage, which may contribute to divergent clinical outcomes. Although subgroup analyses were conducted based on PRP preparation methods, total blood volume, and injection volume, the limited number of studies and small sample sizes necessitate cautious interpretation of these findings. Finally, the follow-up duration in all included studies was ≤12 months, highlighting the necessity for further studies with extended follow-up periods and larger sample sizes. Future research should standardize PRP preparation methods, injection protocols, and follow-up timepoints to allow more robust comparisons across studies.

## Conclusions

Compared to patients undergoing PELD alone, the combination of PELD and PRP injection therapy demonstrated positive effects in alleviating pain and improving function. Subgroup analysis at different follow-up time points also revealed improved clinical outcomes with the combined therapy. Due to the limited number of included studies, it is necessary to evaluate the long-term clinical efficacy and safety of this combined therapy by increasing the sample size and extending the follow-up duration.

## Data Availability

The original contributions presented in the study are included in the article/Supplementary Material, further inquiries can be directed to the corresponding author.

## References

[1] Zhang AS Xu A Ansari K Hardacker K Anderson G Alsoof D Lumbar disc herniation: diagnosis and management. Am J Med. (2023) 136(7):645–51. 10.1016/j.amjmed.2023.03.024 37072094

[2] Pojskic M Bisson E Oertel J Takami T Zygourakis C Costa F. Lumbar disc herniation: epidemiology, clinical and radiologic diagnosis WFNS spine committee recommendations. World Neurosurg X. (2024) 22:100279. 10.1016/j.wnsx.2024.100279 38440379 PMC10911853

[3] Chu EC Sabourdy E. Non-surgical restoration of L3/L4 disc herniation. Cureus. (2023) 15(6):e40941. 10.7759/cureus.40941 37496528 PMC10368486

[4] Lee YC Zotti MG Osti OL. Operative management of lumbar degenerative disc disease. Asian Spine J. (2016) 10(4):801–19. 10.4184/asj.2016.10.4.801 27559465 PMC4995268

[5] Deyo RA Mirza SK Martin BI Kreuter W Goodman DC Jarvik JG. Trends, major medical complications, and charges associated with surgery for lumbar spinal stenosis in older adults. JAMA. (2010) 303(13):1259–65. 10.1001/jama.2010.338 20371784 PMC2885954

[6] Suwa H Hanakita J Ohshita N Gotoh K Matsuoka N Morizane A. Postoperative changes in paraspinal muscle thickness after various lumbar back surgery procedures. Neurol Med Chir. (2000) 40(3):151–4; discussion 4–5. 10.2176/nmc.40.151 10842484

[7] Wang A Yu Z. Comparison of percutaneous endoscopic lumbar discectomy with minimally invasive transforaminal lumbar interbody fusion as a revision surgery for recurrent lumbar disc herniation after percutaneous endoscopic lumbar discectomy. Ther Clin Risk Manag. (2020) 16:1185–93. 10.2147/TCRM.S283652 33363376 PMC7754645

[8] Sharma M Chhawra S Jain R Sharma S. Full endoscopic lumbar transforaminal interbody fusion in DDD lumbar degenerative disc disease: a latest technique. Int J Spine Surg. (2021) 14(s4):S71–s7. 10.14444/7168 33900948 PMC7888205

[9] Pan M Li Q Li S Mao H Meng B Zhou F Percutaneous endoscopic lumbar discectomy: indications and complications. Pain Physician. (2020) 23(1):49–56. 32013278

[10] Zhang B Dong B Wang L Wang Y Gao Z Li Y Comparison of the efficacy of autologous Lp-PRP and Lr-PRP for treating intervertebral disc degeneration: *in vitro* and *in vivo* study. J Orthop Surg Res. (2024) 19(1):731. 10.1186/s13018-024-05196-8 39506797 PMC11542231

[11] Jayasoorya A Samal N Pisulkar G Datta K Kawde K. Injections of platelet-rich plasma: an emerging novel biological cure for low back pain? Cureus. (2024) 16(2):e54048. 10.7759/cureus.54048 38481898 PMC10934062

[12] Beall DP Kim KD Macadaeg K Donboli K Chauhan K Sowlay M Treatment gaps and emerging therapies in lumbar disc herniation. Pain Physician. (2024) 27(7):401–13. 39353108

[13] Wang SZ Chang Q Lu J Wang C. Growth factors and platelet-rich plasma: promising biological strategies for early intervertebral disc degeneration. Int Orthop. (2015) 39(5):927–34. 10.1007/s00264-014-2664-8 25653173

[14] Wongjarupong A Pairuchvej S Laohapornsvan P Kotheeranurak V Jitpakdee K Yeekian C “Platelet-Rich Plasma” epidural injection an emerging strategy in lumbar disc herniation: a randomized controlled trial. BMC Musculoskelet Disord. (2023) 24(1):335. 10.1186/s12891-023-06429-3 37118707 PMC10141936

[15] Lana J da Fonseca LF Macedo RDR Mosaner T Murrell W Kumar A Platelet-rich plasma vs bone marrow aspirate concentrate: an overview of mechanisms of action and orthobiologic synergistic effects. World J Stem Cells. (2021) 13(2):155–67. 10.4252/wjsc.v13.i2.155 33708344 PMC7933989

[16] Mohammed S Yu J. Platelet-rich plasma injections: an emerging therapy for chronic discogenic low back pain. J Spine Surg. (2018) 4(1):115–22. 10.21037/jss.2018.03.04 29732431 PMC5911760

[17] Page MJ McKenzie JE Bossuyt PM Boutron I Hoffmann TC Mulrow CD The PRISMA 2020 statement: an updated guideline for reporting systematic reviews. Br Med J. (2021) 372:n71. 10.1136/bmj.n71 33782057 PMC8005924

[18] Moher D Liberati A Tetzlaff J Altman DG. Preferred reporting items for systematic reviews and meta-analyses: the PRISMA statement. Open Med. (2009) 3(3):e123–30. 21603045 PMC3090117

[19] Higgins JP Altman DG Gøtzsche PC Jüni P Moher D Oxman AD The cochrane collaboration’s tool for assessing risk of bias in randomised trials. Br Med J. (2011) 343:d5928. 10.1136/bmj.d5928 22008217 PMC3196245

[20] Shim SR Kim SJ. Intervention meta-analysis: application and practice using R software. Epidemiol Health. (2019) 41:e2019008. 10.4178/epih.e2019008 30999738 PMC6545497

[21] Higgins JP Thompson SG. Quantifying heterogeneity in a meta-analysis. Stat Med. (2002) 21(11):1539–58. 10.1002/sim.1186 12111919

[22] Li T Du W Ding Z Liu J Ding Y. Percutaneous endoscopic lumbar discectomy combined with platelet-rich plasma injection for lumbar disc herniation: analysis of clinical and imaging outcomes. BMC Musculoskelet Disord. (2024) 25(1):328. 10.1186/s12891-024-07444-8 38658984 PMC11044406

[23] Zhang L Zhang C Song D Chen G Liu L. Combination of percutaneous endoscopic lumbar discectomy and platelet-rich plasma hydrogel injection for the treatment of lumbar disc herniation. J Orthop Surg Res. (2023) 18(1):609. 10.1186/s13018-023-04093-w 37605261 PMC10440935

[24] Jiang Y Zuo R Yuan S Li J Liu C Zhang J Transforaminal endoscopic lumbar discectomy with versus without platelet-rich plasma injection for lumbar disc herniation: a prospective cohort study. Pain Res Manag. (2022) 2022:6181478. 10.1155/2022/6181478 35296040 PMC8920626

[25] Qi H Zhou Z Li G Huang Y Chen S Liu B. Evaluating the impact of platelet-rich plasma injection in spinal endoscopic nucleotomy on MRI pfirrmann grading and clinical outcomes in lumbar disc herniation. J Orthop Surg Res. (2024) 19(1):655. 10.1186/s13018-024-05153-5 39402583 PMC11476116

[26] Dhurat R Sukesh M. Principles and methods of preparation of platelet-rich plasma: a review and Author’s perspective. J Cutan Aesthet Surg. (2014) 7(4):189–97. 10.4103/0974-2077.150734 25722595 PMC4338460

[27] Everts P Onishi K Jayaram P Lana JF Mautner K. Platelet-rich plasma: new performance understandings and therapeutic considerations in 2020. Int J Mol Sci. (2020) 21(20):7794. 10.3390/ijms21207794 33096812 PMC7589810

[28] Akeda K Ohishi K Masuda K Bae WC Takegami N Yamada J Intradiscal injection of autologous platelet-rich plasma releasate to treat discogenic low back pain: a preliminary clinical trial. Asian Spine J. (2017) 11(3):380–9. 10.4184/asj.2017.11.3.380 28670405 PMC5481592

[29] Akeda K Takegami N Yamada J Fujiwara T Ohishi K Tamaru S Platelet-rich plasma-releasate (PRPr) for the treatment of discogenic low back pain patients: long-term follow-up survey. Medicina. (2022) 58(3):428. 10.3390/medicina58030428 35334604 PMC8952290

[30] Cheng J Santiago KA Nguyen JT Solomon JL Lutz GE. Treatment of symptomatic degenerative intervertebral discs with autologous platelet-rich plasma: follow-up at 5–9 years. Regen Med. (2019) 14(9):831–40. 10.2217/rme-2019-0040 31464577 PMC6770415

[31] Muthu S Jeyaraman M Chellamuthu G Jeyaraman N Jain R Khanna M. Does the intradiscal injection of platelet rich plasma have any beneficial role in the management of lumbar disc disease? Global Spine J. (2022) 12(3):503–14. 10.1177/2192568221998367 33840260 PMC9121148

[32] Centeno C Markle J Dodson E Stemper I Hyzy M Williams C The use of lumbar epidural injection of platelet lysate for treatment of radicular pain. J Exp Orthop. (2017) 4(1):38. 10.1186/s40634-017-0113-5 29177632 PMC5701904

[33] Le V-T Nguyen Dao LT Nguyen AM. Transforaminal injection of autologous platelet-rich plasma for lumbar disc herniation: a single-center prospective study in Vietnam. Asian J Surg. (2023) 46(1):438–43. 10.1016/j.asjsur.2022.05.047 35637114

[34] Jain D Goyal T Verma N Paswan AK Dubey RK. Intradiscal platelet-rich plasma injection for discogenic low back pain and correlation with platelet concentration: a prospective clinical trial. Pain Med. (2020) 21(11):2719–25. 10.1093/pm/pnaa254 32869064

[35] Bise S Dallaudiere B Pesquer L Pedram M Meyer P Antoun MB Comparison of interlaminar CT-guided epidural platelet-rich plasma versus steroid injection in patients with lumbar radicular pain. Eur Radiol. (2020) 30(6):3152–60. 10.1007/s00330-020-06733-9 32095875

[36] Mern DS Beierfuβ A Fontana J Thomé C Hegewald AA. Imbalanced protein expression patterns of anabolic, catabolic, anti-catabolic and inflammatory cytokines in degenerative cervical disc cells: new indications for gene therapeutic treatments of cervical disc diseases. PLoS One. (2014) 9(5):e96870. 10.1371/journal.pone.0096870 24804684 PMC4013068

[37] Gullung GB Woodall JW Tucci MA James J Black DA McGuire RA. Platelet-rich plasma effects on degenerative disc disease: analysis of histology and imaging in an animal model. Evid Based Spine Care J. (2011) 2(4):13–8. 10.1055/s-0031-1274752 23230401 PMC3506140

[38] Chen WH Liu HY Lo WC Wu SC Chi CH Chang HY Intervertebral disc regeneration in an ex vivo culture system using mesenchymal stem cells and platelet-rich plasma. Biomaterials. (2009) 30(29):5523–33. 10.1016/j.biomaterials.2009.07.019 19646749

[39] Obata S Akeda K Imanishi T Masuda K Bae W Morimoto R Effect of autologous platelet-rich plasma-releasate on intervertebral disc degeneration in the rabbit anular puncture model: a preclinical study. Arthritis Res Ther. (2012) 14(6):R241. 10.1186/ar4084 23127251 PMC3674597

[40] Li P Zhang R Zhou Q. Efficacy of platelet-rich plasma in retarding intervertebral disc degeneration: a meta-analysis of animal studies. Biomed Res Int. (2017) 2017:7919201. 10.1155/2017/7919201 28752097 PMC5511641

