# Supplementary material for: The clinical efficacy of percutaneous endoscopic lumbar discectomy combined with platelet-rich plasma injection for lumbar disc herniation: a systematic review and meta-analysis
Source: Front Surg. 2025 May 27;12:1601772. doi: 10.3389/fsurg.2025.1601772 (PMC12148837; doi:10.3389/fsurg.2025.1601772)
Supplement: Supplementary file 1 [file Datasheet1.docx]

**Supplementary Materials**


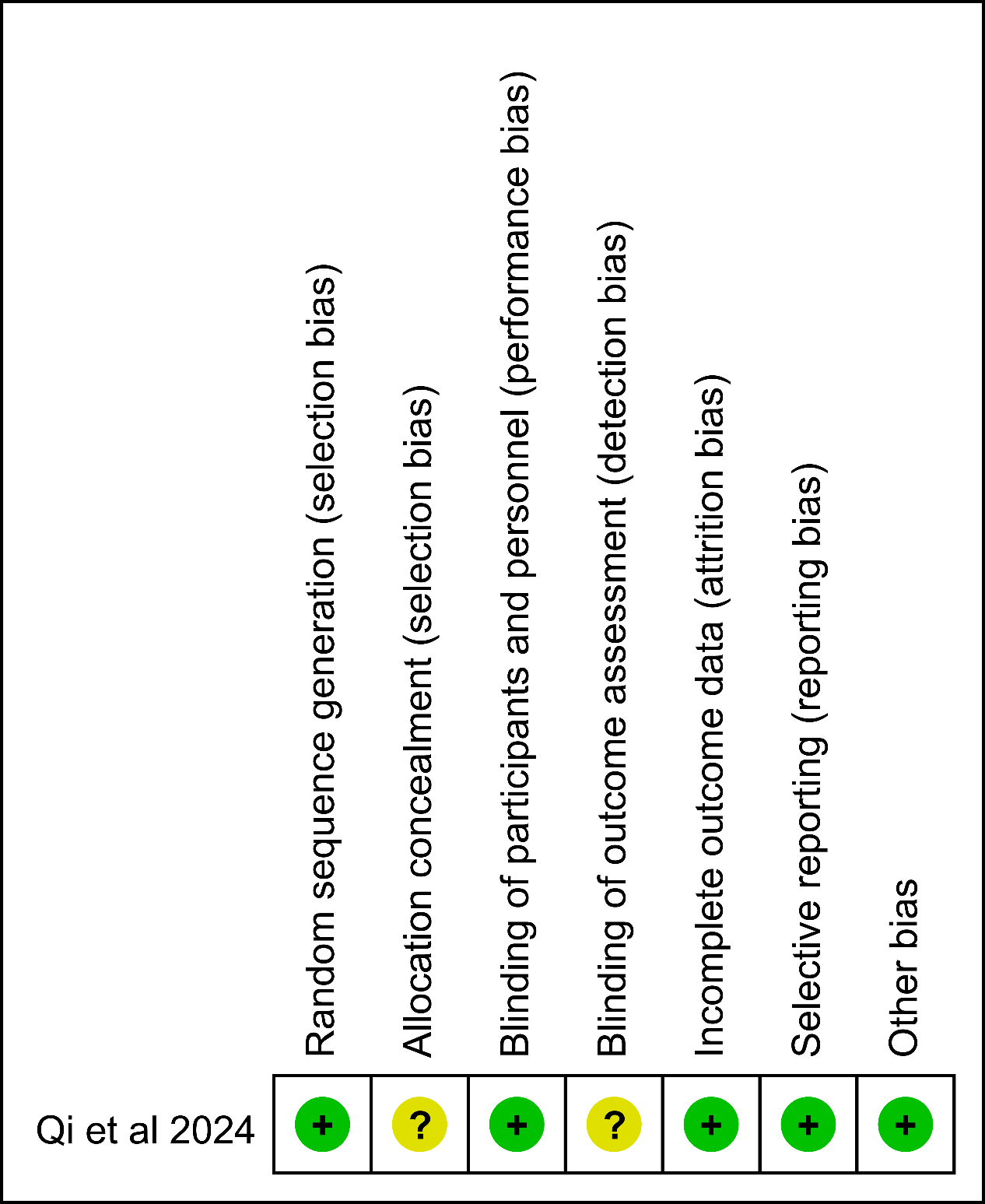


**Supplementary Figure S1.** Risk of bias summary for the RCT.


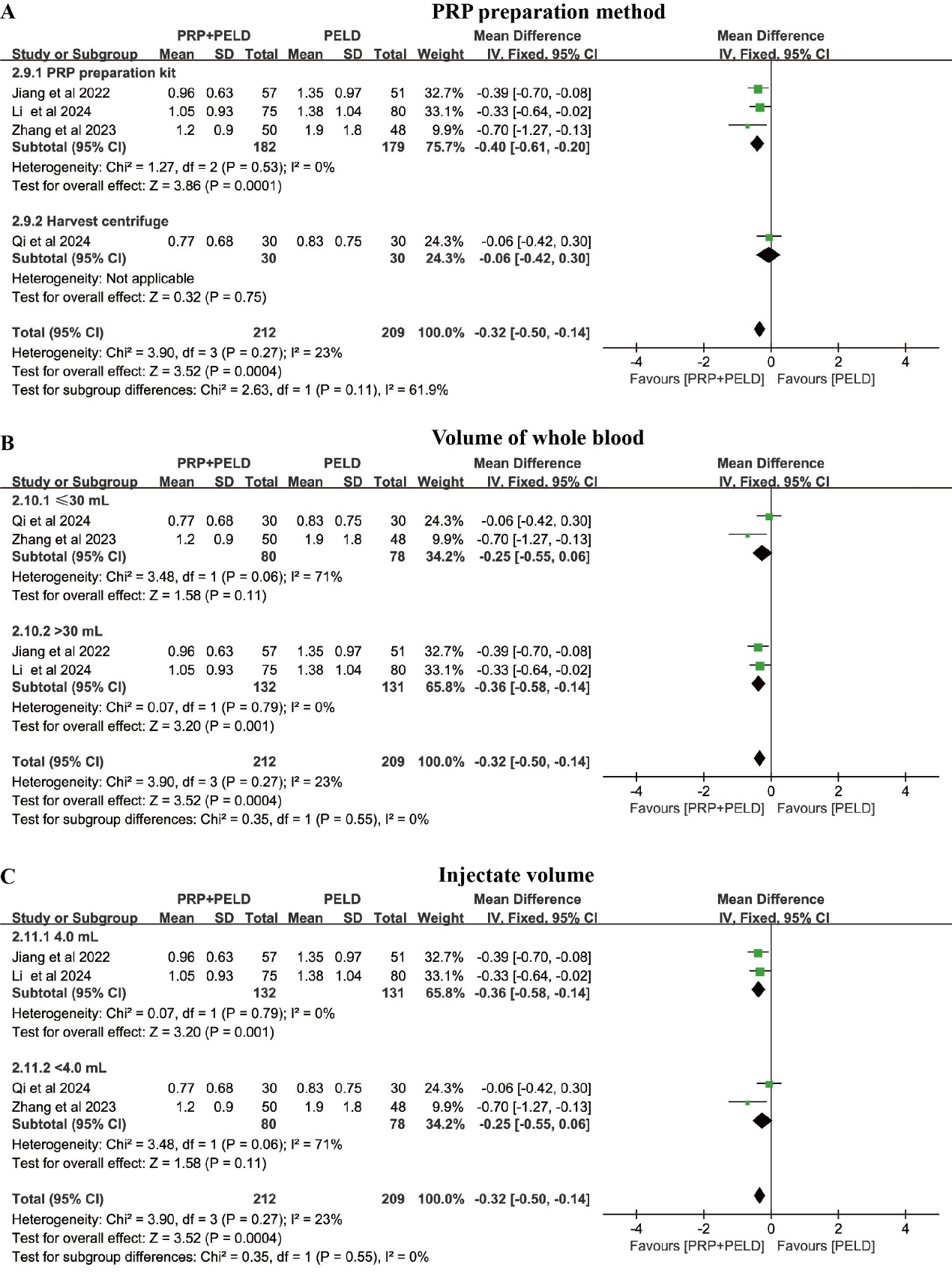


**Supplementary Figure S2.** Subgroup analysis of VAS scores for low back pain at 3 months. (A) Subgroup analysis based on different PRP preparation methods; (B) Subgroup analysis based on volume of whole blood used; (C) Subgroup analysis based on different injectate volumes.


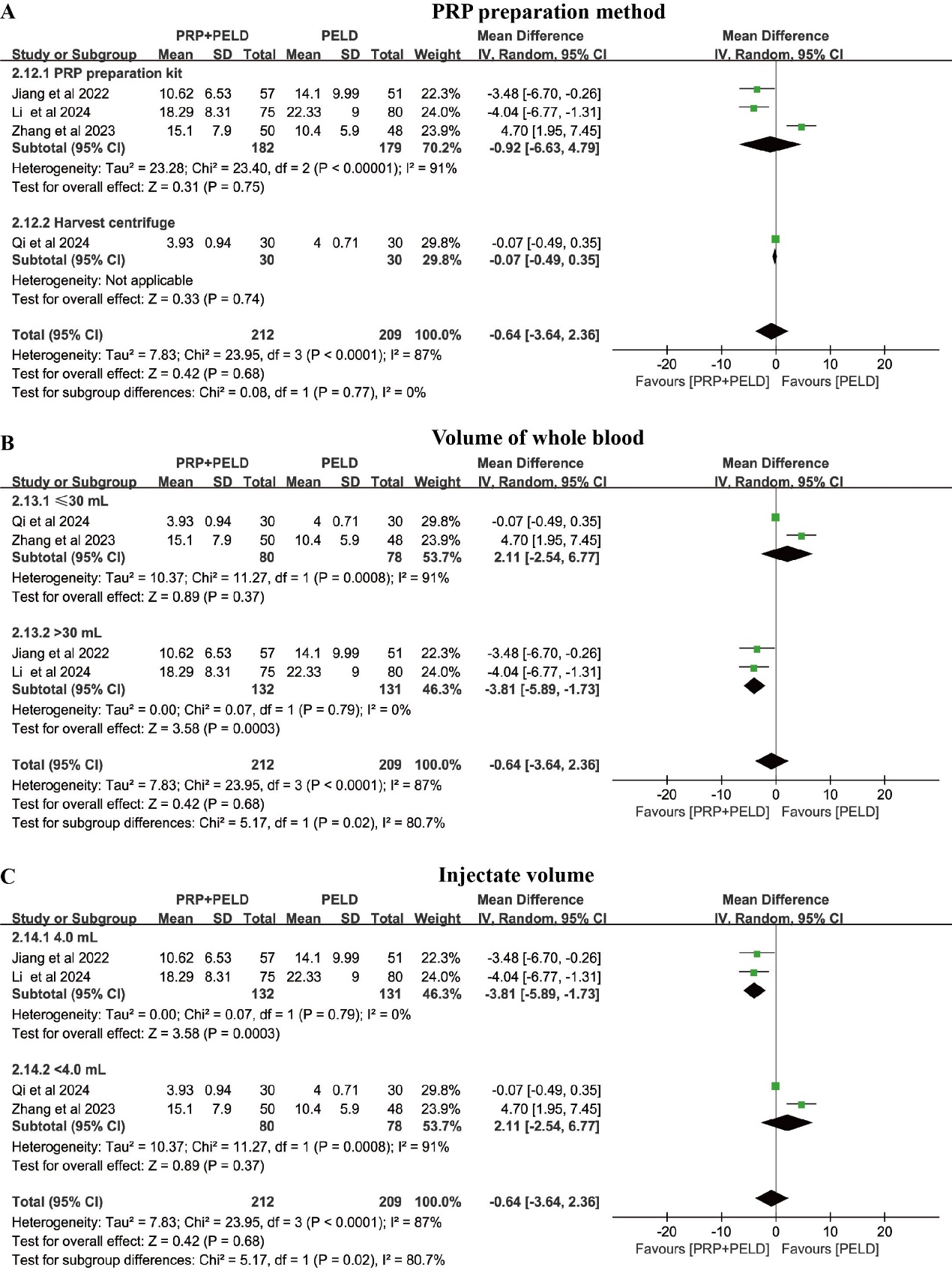


**Supplementary Figure S3.** Subgroup analysis of ODI scores at 3 months. (A) Subgroup analysis based on different PRP preparation methods; (B) Subgroup analysis based on volume of whole blood used; (C) Subgroup analysis based on different injectate volumes.

| **Supplementary Table S1.** Search Strages in Pubmed. | | | |
| --- | --- | --- | --- |
| **Database** | **No.** | **Query** | **Results** |
| **Pubmed** | #1 | "percutaneous endoscopic lumbar discectomy" OR "PELD" OR "endoscopic discectomy" OR "platelet-rich plasma" OR "PRP" OR "platelet concentrates" | 35,077 |
|  | #2 | "lumbar disc herniation" OR "LDH" OR "herniated lumbar disc" OR "lumbar intervertebral disc herniation" | 52,102 |
|  | #3 | #1 AND #2 | 742 |

| **Supplementary Table S2**. Newcastle-Ottawa Scale for risk of bias assessment of cohort studies included in the meta-analysis. | | | | | | | | | |
| --- | --- | --- | --- | --- | --- | --- | --- | --- | --- |
| Study | Selection | | | | Comparability | Exposure | | | Scores |
|  | Representativeness of Exposed Cohort | Selection of Nonexposed | Ascertainment of Exposure | Outcome Not Present at Start | Comparability between groups | Assessment of Outcome | Adequate Follow- Up Length | Adequacy of Follow-Up |  |
| Jiang 2022 | ★ | ★ | ★ | ★ | ★★ | ★ | ★ | ★ | 9 |
| Zhang 2023 | ★ | ★ | ★ | ★ | ★★ | ★ |  | ★ | 8 |
| Li 2024 | ★ | ★ | ★ | ★ | ★★ | ★ | ★ | ★ | 9 |
| ★: score of 1; ★★: score of 2 | | | | | | | | | |
